# The complex interplay between psoriasis and depression: from molecular mechanisms to holistic treatment approaches

**DOI:** 10.3389/fimmu.2025.1659346

**Published:** 2025-11-21

**Authors:** Ying Wang, Jianxiao Xing, Yanyang Liang, Huifang Liang, Xuping Niu, Junqin Li, Kaiming Zhang

**Affiliations:** ShanXi Key Laboratory of Stem Cells for Immunological Dermatosis, State Key Breeding Laboratory of Stem Cells for Immunological Dermatosis, Institute of Dermatology, Taiyuan Central Hospital of Shanxi Medical University, Taiyuan, China

**Keywords:** psoriasis, depression, comorbidity, mechanism, treatment

## Abstract

Psoriasis is an inflammatory disease driven by immune dysregulation. Numerous epidemiological studies have confirmed a significant association between psoriasis and mental health disorders, particularly depression. Recent research has increasingly underscored the common pathogenic mechanisms between psoriasis and depression. The release of factors such as TNF, IL-6, and IL-8 not only directly drives abnormal proliferation of skin keratinocytes and immune infiltration but also disrupts the blood-brain barrier, inducing neuroinflammation. Hypothalamic-pituitary-adrenal (HPA) axis dysfunction leads to inflammation amplification. Additionally, microbiota dysbiosis—such as a reduced abundance of Actinobacteria and Firmicutes—decreases the production of short-chain fatty acids and increases the absorption of lipopolysaccharides into the bloodstream via the “gut-brain-skin axis,” thereby exacerbating systemic and neuroinflammatory conditions. Based on this understanding, clinical practice demands an integrated “biological-psychological-social” approach. Biologics (e.g., adalimumab, secukinumab, and guselkumab) can simultaneously ameliorate skin lesions and depressive symptoms. When combined with psychological interventions—including cognitive behavioral therapy and mindfulness therapy—a multidisciplinary collaboration involving dermatology, rheumatology, and psychology is essential to formulate a tailored treatment plan. This review systematically outlines the core mechanisms underlying comorbidity of these two diseases, as well as multidimensional treatment strategies.

## 1 Introduction

Psoriasis is an inflammatory immune-mediated dermatosis characterized by excessive proliferation of keratinocytes, manifesting clinically as erythematous squamous plaques on the trunk, limbs, scalp, and facial regions. Despite advancements in our understanding of its pathogenesis, the precise etiology of psoriasis remains incompletely elucidated. Psoriasis significantly impacts patients’ quality of life, with burden metrics comparable to malignancies, chronic heart failure, and chronic obstructive pulmonary disease, as it contributes to disability, premature mortality, and economic strain on social and public health systems (1, 2). In recent years, the comorbidity of psoriasis and depression has garnered substantial attention (3, 4). Depression, a chronic mental disease characterized by high incidence, disability and mortality rates, exacerbates the psychological burden experienced by psoriasis patients due to itching, pain, and disfiguring lesions (5, 6). According to a study by Luna et al., the prevalence of psoriasis comorbid with depression ranges from 0.2% to 74.6%, and the incidence rate is (4.83–91.9) per 1,000 person-years. Multiple studies have confirmed that patients with psoriasis are prone to comorbid major depression, and the risk of depression in patients with severe psoriasis is higher than that in those with mild psoriasis (7, 8). Notably, depression increases the risk of psoriasis recurrence by 1.5 times and predisposes patients to a more severe disease course.

In psoriasis patients, depressive symptoms frequently culminate in psychological disability and therapeutic resistance, hindering optimal therapeutic outcomes. The interplay between psoriasis and depression is multifaceted, involving shared pathogenesis, clinical manifestations, and treatment management. Moreover, depression is not merely a psychological complication of psoriasis; it may also exacerbate skin inflammation via neuroimmune mechanisms, creating a vicious cycle (9, 10). This article aims to provide a comprehensive review encompassing multiple dimensions, including epidemiology, pathological mechanisms, clinical impact, and treatment strategies, based on recent research advancements.

## 2 Pathogenetic interaction between psoriasis and depression

### 2.1 Genetic factors

Psoriasis and depression exhibit a significant genetic association. Studies investigating familial patterns of both conditions have identified chromosomes 8, 22, 21, 15, X, and Y as significantly implicated in their co-occurrence (11). Psoriasis is recognized as a polygenic disease, and multiple genetic susceptibility genes can contribute to its onset through various mechanisms, including immune responses, infections, and emotional factors. Among these susceptibility genes, the human leukocyte antigen class I allele HLA-C*06:02 has been identified as a key risk gene for the development of psoriasis (12). This gene may facilitate the onset of psoriasis through the presentation of self-antigens by skin-specific cell populations. Zhang Qiang et al. demonstrated that negative emotions interact with the REL gene polymorphism (rs702873) and the TNF-α-induced protein 3-interacting protein 1 (TNIP1) gene polymorphism (rs17728338), resulting in increased nuclear factor kappa B (NF-κB) expression (13). NF-κB is a crucial signaling pathway in the pathogenesis of psoriasis, playing a significant role in inducing the expression and release of pro-inflammatory cytokines. Mutations in psoriasis-related genes can lead to the excessive release of pro-inflammatory cytokines, which in turn excessively activate the hypothalamic-pituitary-adrenal (HPA) axis and reduce the level of serotonin neurotransmitter, ultimately resulting in the onset of depression. Additionally, evidence indicates that the risk of severe depression is threefold higher in first-degree relatives of psoriasis patients compared to those without a familial history of severe depression, further supporting the correlation between psoriasis and depression (14). Furthermore, genetic and environmental factors jointly drive the development of psoriasis and depression. Environmental factors can directly regulate gene expression through epigenetic mechanisms, triggering the activation of specific genes. This process is called gene-environment interaction, which is controlled by epigenetic mechanisms (15). In a study of saliva samples from students with depression, self-harm behaviors, and suicidal ideation, differentially methylated genes were significantly enriched in immune response pathways, with psoriasis-related genes being particularly prominent. These include members of the LCE family and novel targets such as MIR4520A/B. Further research shows that LCE family genes are densely located in the epidermal differentiation complex (EDC) region of chromosome 1q21.3, which also contains multiple skin-surface-specific expressed genes—suggesting that the immune-epidermal regulatory network may be involved in the pathological process of depression (16). Concurrently, an independent epigenetic screening by Murphy and colleagues revealed a molecular link between psoriasis and depression. By comparing methylation patterns in brain regions BA11 and BA25 of suicidal depression patients and normal controls, the study confirmed that methylation levels of the psoriasis susceptibility gene PSORS1C3 were significantly reduced in the suicidal depression group, a phenomenon not observed in controls. This finding highlights the potential association between PSORS1C3 methylation status and suicidal behavior in depressed patients (17). These studies not only provide new insights into the shared molecular mechanisms of depression and psoriasis but also lay a theoretical foundation for developing therapeutic strategies for the comorbidity of these two diseases.

### 2.2 Neuro-endocrine-immune network

The hypothalamic-pituitary-adrenal axis (HPA axis), as the core regulatory pathway of the neuro-immune-endocrine system, plays a crucial role in the mechanism underlying the comorbidity of psoriasis and depression. Under normal physiological conditions, the HPA axis maintains stable hormone levels through a sophisticated negative feedback mechanism: the hypothalamus secretes corticotropin-releasing hormone (CRH), which acts on the anterior pituitary to stimulate the release of adrenocorticotropic hormone (ACTH); ACTH is transported via the bloodstream to the adrenal cortex, where it stimulates the synthesis and secretion of glucocorticoids (e.g., cortisol); finally, cortisol binds to glucocorticoid receptors (GR) in the hypothalamus, pituitary gland, and hippocampus, inhibiting the excessive secretion of CRH and ACTH, thus forming a complete regulatory loop to maintain stable hormone levels. As a chronic inflammatory skin disease, psoriasis-induced peripheral disturbance to the HPA axis is mainly reflected in the abnormal local inflammatory microenvironment of the skin and the dysfunction of the “peripheral HPA axis”, which is also the primary link impairing the function of the central HPA axis. The skin, as a “peripheral endocrine organ”, can independently synthesize CRH, ACTH, and cortisol, forming a “local HPA axis” independent of the central nervous system. The dysfunction of this local axis directly contributes to the pathogenesis of psoriasis: keratinocytes, fibroblasts, mast cells, and other cells in psoriatic lesions secrete large amounts of CRH (18). By binding to the highly expressed CRH1 receptors (CRHR1) on the cell surface—with CRHR1 expression in lesional skin significantly higher than in normal skin—CRH further stimulates the release of pro-inflammatory factors such as IL-6, IL-22, and VEGF (18, 19), exacerbating skin inflammation and forming a positive feedback loop of “CRH-CRHR1-inflammation” (19, 20). One of the core pathological mechanisms of depression is “central neuroinflammation”. Its impact on the HPA axis is mainly manifested as “failure of central negative feedback inhibition”, which in turn leads to sustained hyperactivity of the HPA axis, further exacerbating neuroinflammation, and forming a “cross-amplification” effect with the peripheral inflammation of psoriasis (21, 22). In depression patients, there is excessive activation of microglia in emotion-regulating brain regions such as the hippocampus, amygdala, and prefrontal cortex, which release pro-inflammatory factors including IL-1β, IL-6, and TNF-α. These central inflammatory factors directly disrupt the negative feedback mechanism of the HPA axis. Among these brain regions, the amygdala (the emotional stress center) is overactivated due to inflammation and directly stimulates hypothalamic CRH neurons through neural circuits, further promoting HPA axis activation and forming a cycle of “amygdala inflammation → HPA axis hyperactivity → emotional disorders” (23, 24). Eventually, psoriasis and depression form a bidirectional pathological cycle of “peripheral inflammation → central HPA axis dysfunction → neuroinflammation → emotional disorders → exacerbation of peripheral inflammation” via the HPA axis. Additionally, chronic stress associated with depression can trigger the release of norepinephrine through the sympathetic nervous system, which in turn stimulates the secretion of IL-17, thereby worsening psoriatic lesions. Ultimately, psoriasis patients with comorbid depression experience prolonged lesion remission times and reduced response rates to biological agents. Furthermore, stress, as a common trigger for both diseases, synchronously activates the HPA axis and inflammatory responses-stress exacerbates skin inflammation in psoriasis and aggravates the failure of HPA axis negative feedback in depression, ultimately forming a vicious cycle.

### 2.3 Neuropeptides and biochemical factors

In the skin, keratinocytes play a critical role in the secretion of various neuropeptides that serve as important links between peripheral and central inflammation. For instance, substance P (SP) and nerve growth factor (NGF) primarily activate the SP neurokinin-1 (NK1) receptor pathway, stimulating the proliferation and infiltration of inflammatory cells (e.g., T lymphocytes) and inducing mast cell degranulation to release cytokines such as IL-12, IL-1, and IL-6 (25). These processes significantly contribute to the inflammatory response observed in conditions such as psoriasis. Vitamin D plays a uniquely significant role in skin health and the management of skin-related diseases. Studies demonstrate that vitamin D not only suppresses the inflammatory response in psoriasis but also aids in rectifying the abnormal epidermal function associated with the disease (26). Furthermore, vitamin D deficiency has been linked to an increased risk of disease, with both psoriasis and depression showing a strong association with vitamin D insufficiency (27, 28). Insufficient vitamin D can exacerbate skin inflammation while also inhibiting the synthesis of serotonin (29). As a key neurotransmitter, serotonin is frequently implicated in the onset of depressive symptoms—impaired synthesis of this molecule often contributes to such symptoms (30). Additionally, reduced melatonin levels may disrupt sleep and emotional homeostasis, further worsening outcomes for patients with comorbid psoriasis and depression (31).

### 2.4 Immune cells and inflammatory factors

Currently, increasing evidence acknowledges that psoriasis and depression may share common inflammatory pathways, with both conditions closely linked to immune system activation and dysregulated inflammatory cytokine secretion. Studies have shown that CD2^+^, CD4^+^, and CD8^+^ T lymphocyte levels are elevated in both patients with depression and psoriatic patients (32). Additionally, the number of Th17 cells and IL-17 levels in psoriatic skin lesions and serum have been positively correlated with psoriasis severity and the risk of developing depression or anxiety (33). Psoriasis-associated inflammatory cytokines (e.g., TNF-α, IL-17A, IL-23) and Th17 cells can compromise the blood-brain barrier (BBB). Disruption of the BBB may lead to reduced concentrations of central neurotransmitter metabolites and impaired neurotransmitter activity, further exacerbating neuropsychiatric symptoms. For instance, TNF-α can activate microglia, inducing excessive glutamate secretion, which may cause neuronal excitotoxic damage, thereby promoting depressive pathogenesis (34, 35). These findings underscore the complex interplay between immune cells, inflammatory factors, and the neurological outcomes observed in psoriasis and depression, highlighting the need for an integrated approach to understanding these interconnected conditions.

### 2.5 Gut microbiota and the gut-brain-skin axis

The gut microbiota is closely associated with human health and disease, playing a key role in maintaining host immune homeostasis. Once microbial homeostasis is disrupted-leading to gut dysbiosis-it may trigger immune disorders and induce inflammatory responses (36–39). In recent years, a growing body of research has focused on the gut microbiota, suggesting it may serve as a potentially important link between psoriasis and depression (40, 41). Research has identified significant alterations in the gut microbiota composition of patients with psoriasis: the relative abundances of the phyla Actinobacteria and Firmicutes are notably reduced, alongside decreased abundances of beneficial bacteria such as Fecalibacterium, Ruminococcus, and Bacteroides (42). These changes indicate that gut microbiota abnormalities play a crucial role in the pathological mechanisms of psoriasis (43). The proposal of the “gut-skin axis” concept further connects gut microbiota shifts to cutaneous inflammatory responses, offering a new perspective for understanding psoriasis pathogenesis (44). In the field of depression, studies have similarly documented compositional and functional changes in patients’ gut microbiota, primarily characterized by reduced microbial diversity and richness (41, 45). Analysis of fecal samples from depressed patients reveals a higher proportion of the phylum Bacteroidetes and a lower proportion of Firmicutes compared to healthy controls (46). Gut-brain communication relies on the “gut-brain axis,” which transmits information via multiple pathways—including neuroendocrine, neuroimmune, and sensory neural pathways—thereby influencing brain function and emotional states (47). Drugs exert a significant impact on the composition and function of the gut microbiota. Currently, biologic immunotherapies dominate psoriasis treatment, and clinical studies have found that different biologics induce specific changes in gut microbial diversity (48). For instance, the use of IL-23 inhibitors (e.g., guselkumab) increases the relative abundances of Roseburia, Lachnoclostridium, Bacteroides vulgatus, Anaerostipes, and Escherichia-Shigella; in contrast, the use of IL-17 inhibitors (e.g., secukinumab or ixekizumab) significantly elevates levels of Bacteroides stercoris and Parabacteroides merdae while reducing Blautia and Roseburia (49–51). These shifts in microbial diversity may regulate intestinal inflammatory responses, thereby affecting psoriasis severity (49–51). Additionally, research shows that compared to untreated psoriasis patients, those with psoriasis successfully treated with ustekinumab exhibit higher proportions of Firmicutes, Pantoea, and unclassified Comamonadaceae, but lower proportions of Actinobacteria, Corynebacterium, Hydrogenimonadaceae, and Streptococcus (52). These bacteria produce butyric acid and medium-chain fatty acids (MCFAs) during metabolism, which inhibit intestinal oxidative stress and regulate the balance of Th17/Treg lymphocytes. Thus, changes in gut microbiota abundance help alleviate psoriasis symptoms (53). In depression treatment, probiotics and their metabolites show promising application prospects. They can improve and maintain the host’s physiological and psychological states through multiple mechanisms—such as regulating neurotransmitter systems, participating in immune pathways, stimulating the enteric nervous system, and balancing gut microecology—making them potential effective measures for preventing and alleviating depression (54, 55). Furthermore, both psoriasis and depression induce gut dysbiosis, which reduces short-chain fatty acids (SCFAs). Decreased SCFAs increase intestinal permeability and bacterial translocation (i.e., “leaky gut”). Lipopolysaccharides (LPS) then enter the bloodstream, where they are recognized by Toll-like receptors, leading to elevated levels of proinflammatory cytokines such as IL-1β, IL-6, TNF-α, and IFN-γ. These cytokines stimulate Th17 cell differentiation and further exacerbate inflammation in both psoriasis and depression. Psoriasis and depression can each disrupt the gut microbiota via the gut-skin axis and gut-brain axis, respectively; in turn, gut dysbiosis amplifies inflammatory responses in both conditions. The “gut-brain-skin axis” model has been proposed to explain the interrelationships among gut microbiota, emotional states, and systemic/cutaneous inflammation (56, 57). This axis may be linked to the overlapping mechanisms of psoriasis and depression. Overall, dysfunction of the gut-brain-skin axis perpetuates a vicious cycle between psoriasis and depression (58, 59).

**Figure 1:**
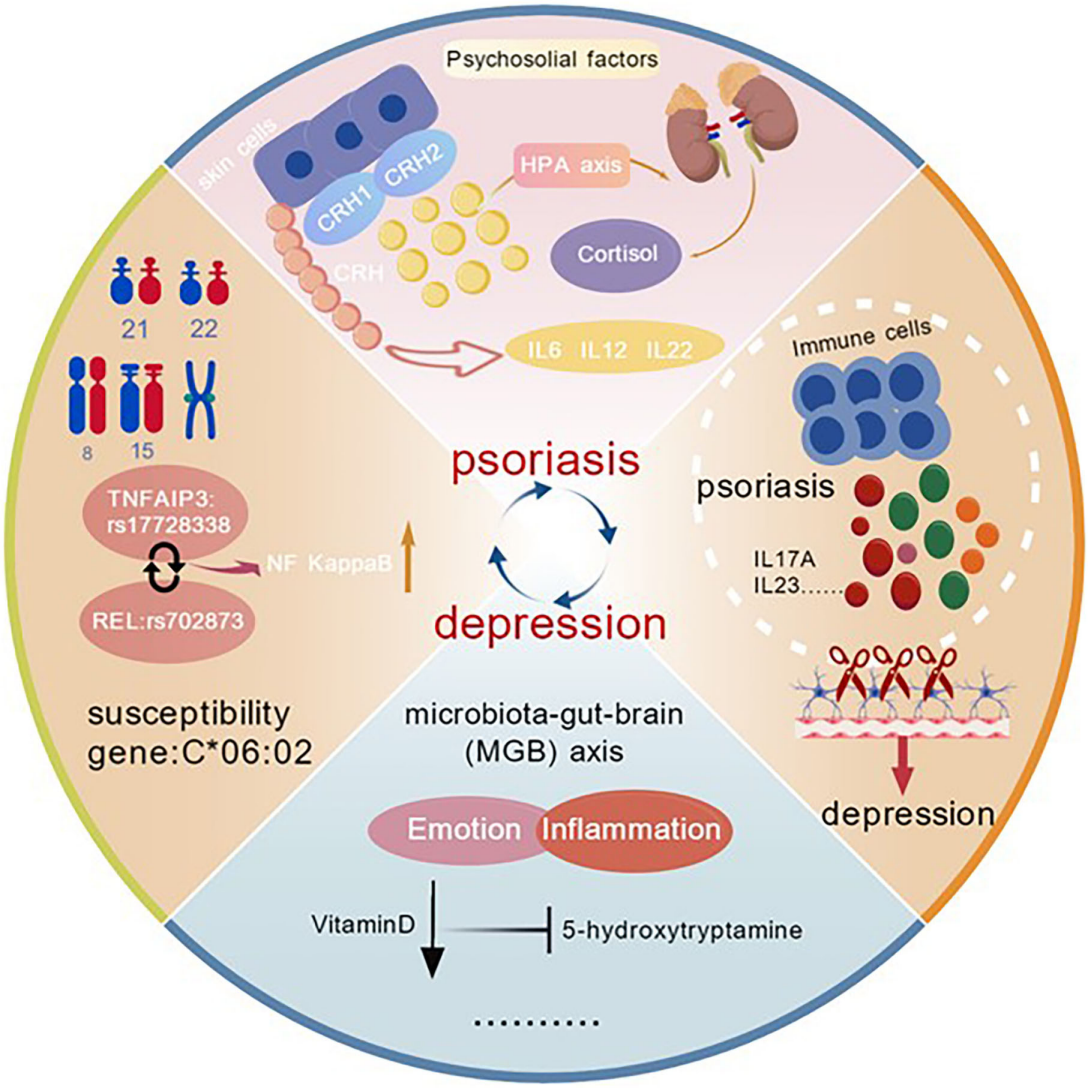
The interactive mechanisms between psoriasis and depression. The interactive mechanisms between the two diseases encompass genetic factors, the neuroendocrine-immune network, neuropeptides, biochemical factors, and psychosocial influences.

The interactive mechanisms between psoriasis and depression are shown in **Figure 1**.

## 3 Assessment of depression in psoriasis patients

The diagnosis of depression in patients with psoriasis primarily relies on dermatologists’ observation of clinical symptoms, as well as patients’ medical and family histories. Additionally, standardized questionnaires such as the Patient Health Questionnaire (PHQ-9) and the Hospital Anxiety and Depression Scale (HADS) are commonly used for assessment (58, 59). Other depression assessment tools—including the Beck Depression Inventory (BDI), the Hamilton Depression Rating Scale (HAM-D), and criteria from the Diagnostic and Statistical Manual of Mental Disorders (DSM)—may also be utilized (60, 61).

Due to variations in screening methods, the reported prevalence of depression among patients with psoriasis varies significantly. For instance, a study by Ye et al. demonstrated that the Chinese Patient Health Questionnaire-9 (C-PHQ-9) is easy to administer for psoriasis patients and has high sensitivity and specificity, thereby serving as a reliable tool for the preliminary screening for depression in this population (62). Furthermore, research by Saito et al. shows that the International Classification of Diseases, 10th edition (ICD-10) is more effective in identifying mild depressive symptoms, whereas the Diagnostic and Statistical Manual of Mental Disorders (DSM, e.g., DSM-5) is better suited for detecting moderate-to-severe depressive symptoms (63–65).

## 4 Comprehensive treatment strategy

### 4.1 Biological agents

Biologics are emerging as a promising therapeutic option for psoriasis, particularly in patients with concurrent depression. A recent prospective case-control study evaluated the therapeutic effects of seven types of biologics in psoriasis patients with depression, revealing that biologic therapy not only alleviates psoriasis severity but also reduces symptoms of depression and anxiety (66). Currently, the biologics approved for psoriasis treatment can be categorized into three main groups: TNF-α inhibitors (including etanercept, infliximab, and adalimumab), IL-12/23 inhibitors (such as ustekinumab and guselkumab), IL-17A inhibitors (such as secukinumab and ixekizumab). Among these biologics, adalimumab (67), ustekinumab (68), and secukinumab (69) have shown considerable efficacy in improving skin lesions and mitigating depressive symptoms in patients with moderate-to-severe psoriasis. Gordon et al. (70) found that guselkumab has superior efficacy compared with adalimumab in improving anxiety and depression symptoms, while also exhibiting sustained clinical benefits over time. Although existing literature suggests that reducing systemic inflammation helps alleviate negative emotional states, it remains unclear whether these effects are primarily due to the direct anti-inflammatory effects of biologics or the resultant improvement in skin lesions and overall quality of life. Thus, while biologics offer a promising avenue for addressing psoriasis-depression comorbidity, there is an urgent need for large-scale, controlled intervention studies to further confirm their safety and efficacy in this context.

### 4.2 Psychotropic drugs therapy

In the context of treating psoriasis comorbid with depression, psychopharmacological therapy has attracted growing attention as a key intervention. A double-blind, placebo-controlled study involving 60 psoriasis patients revealed that the treatment group receiving a combination of antidepressants (e.g., sertraline—a selective serotonin reuptake inhibitor) and topical corticosteroids experienced more pronounced improvements in skin lesions and greater alleviation of depressive and anxiety symptoms, compared with other treatment approaches (71). Furthermore, a clinical study of 38 psoriasis patients found that escitalopram (a selective serotonin reuptake inhibitor, SSRI) combined with psychological interventions resulted in more significant improvements in skin lesions, disease-related cognition, quality of life, and treatment adherence among psoriatic patients with mood disorders (72). Despite these promising findings, clinical evidence supporting the use of psychotropic drugs for this comorbidity remains limited. Moreover, certain antidepressants—such as fluoxetine and bupropion—have been associated with the risk of inducing or exacerbating psoriasis symptoms (73). However, emerging research highlights a close association between psoriasis, depressive states, and neuroinflammation (74). Notably, antidepressants have been shown to significantly reduce peripheral cytokine levels in patients with severe depression, thereby helping to mitigate inflammatory responses (75). These findings suggest that psychotropic medications may serve as adjunctive therapies for managing psoriasis-depression comorbidity, offering new possibilities for treating this complex comorbid condition.

### 4.3 Psychological intervention therapy

Psychological stress has been shown to activate the HPA axis and sympathetic nervous system, thereby disrupting immune function and potentially triggering or exacerbating psoriasis and depression (76). Excessive anxiety not only worsens physical discomfort in patients but also impairs self-perception and social functioning, inflicting both physical and psychological harm on individuals with psoriasis. Psychological interventions aim to reduce disease burden by alleviating negative emotions induced by psychological stress. These approaches primarily include cognitive behavioral therapy (CBT), mind-body interventions (MBIs), multidisciplinary team-based care, and patient education (77).

Cognitive behavioral therapy (CBT) is a conventional psychological supportive therapy that helps patients actively identify and modify maladaptive beliefs, as well as adjust negative thought and behavior patterns, thereby reducing psychological distress. In recent years, numerous studies have demonstrated that CBT produces significant benefits: it reduces psoriasis severity, eases stress and negative emotions, and improves quality of life (77, 78). Cuijpers et al. (79) conducted a meta-analysis of 53 randomized controlled trials (RCTs), which showed that CBT is able to reduce the risk of depression relapse by over 50% after discontinuation—particularly in patients with 1–2 prior depressive episodes. Another meta-analysis, involving 52,702 patients, further revealed that while CBT’s short-term efficacy is not significantly different from that of antidepressants, its effects are more durable at the 6–12 month follow-up, with a 35% lower recurrence rate than the medication group (80). Beyond its effects on mood regulation and depression relapse prevention, CBT also alleviates negative emotions triggered by the appearance of skin lesions and treatment-related stress. This, in turn, improves treatment adherence, which indirectly supports psoriasis management by reducing disease severity and enhancing patients’ quality of life. Ultraviolet B (UVB), particularly narrow-band UVB (NB-UVB), exerts a dual regulatory effect on both skin physiology and mood. As a first-line physical therapy for psoriasis, NB-UVB combined with acitretin capsules not only effectively improves lesions in patients with psoriasis vulgaris but also alleviates comorbid anxiety and depression, while demonstrating a favorable safety profile. Furthermore, upon UVB exposure, the skin can convert 7-dehydrocholesterol into vitamin D3. Mechanistically, vitamin D3 promotes serotonin synthesis in the brain by enhancing tryptophan hydroxylase activity and inhibits the release of pro-inflammatory factors (e.g., interleukin-6 [IL-6] and tumor necrosis factor-α [TNF-α]), thereby mitigating neuroinflammation and contributing to depression regulation. Consistently, accumulating studies have confirmed a robust association between UV exposure and mood improvement, with UV radiation shown to alleviate depressive symptoms (81). Given the “skin-psychological” comorbid characteristics of psoriasis and depression, comprehensive intervention regimens are more effective, among which the combined application of UVB and CBT is particularly prominent. A single-blind randomized controlled trial verified this finding: 40 patients with psoriasis were enrolled and randomly divided into two groups. The treatment group received standard NB-UVB phototherapy plus an additional 8-week intervention of CBT combined with biofeedback. In this group, 65% of patients achieved the “PASI75” criterion (≥75% improvement in psoriasis lesion area and severity), which was significantly higher than the control group (treated with NB-UVB alone), where only 15% of patients reached PASI75. This between-group difference was statistically significant (p=0.007), suggesting that this combined approach can significantly enhance the lesion clearance efficacy of NB-UVB (82).Meditation-based mind-body interventions (MBIs), broadly speaking, encompass a class of interventions that integrate physical and mental practices to alleviate psychological stress. Among diverse mind-body approaches, mindfulness meditation has gained widespread clinical application. Stadtmuller et al. (83) found that mindfulness meditation training significantly reduces psoriasis severity and improves patients’ quality of life. Fordham et al. (84) reached similar conclusions but noted no significant difference between conventional treatment combined with mindfulness therapy and conventional treatment alone in alleviating negative emotions among psoriasis patients. In contrast, research by Liao Fangfang yielded opposing results (85). Additionally, additional studies have demonstrated that mindfulness therapy can significantly reduce levels of C-reactive protein (CRP) and inflammatory cytokines, thereby improving patients’ overall inflammatory profiles and enhancing their stress-coping abilities (86).Collectively, these findings highlight the potential of psychological interventions as effective strategies for managing the comorbidity of psoriasis and depression, suggesting that a multidisciplinary approach is likely to yield the most favorable outcomes for patients.

### 4.4 Multidisciplinary diagnosis, treatment and educational intervention

The diagnosis, treatment, and management of patients with psoriasis require collaborative efforts across multiple disciplines, including dermatology, rheumatology, and immunology, as well as psychology and sports nutrition. Such multidisciplinary approaches can significantly improve treatment compliance, reduce treatment costs, and optimize disease outcomes (87). A study by Iuchetui et al. confirms that collaborative care between dermatology and rheumatology-immunology disciplines leads to notable improvements in clinical symptoms and quality of life among patients with psoriatic arthritis (88).

Throughout the care process, it is essential to conduct regular screenings for comorbidities such as cardiovascular disease and metabolic syndrome. Additionally, lifestyle modifications—including smoking cessation and maintaining regular sleep schedules—can help mitigate the systemic inflammatory burden (89). The “treatment pyramid” theory proposed by Peking Union Medical College Hospital emphasizes the importance of integrating basic nursing care with individualized medication regimens, providing scientific guidance for the comprehensive management of psoriasis (90, 91).

Collectively, these findings underscore the necessity of a multidisciplinary strategy for the diagnosis and treatment of psoriasis, while highlighting the role of collaborative care and patient education in enhancing treatment efficacy and promoting patients’ overall well-being.

The comprehensive treatment strategies for psoriasis-depression comorbidity are summarized in **Table 1**.

**Table 1:** The comprehensive treatment strategies for psoriasis-depression comorbidity.

Treatment method		Therapeutic effect
Biological agents	TNF-α inhibitors:Etanercept, Infliximab, Adalimumab; IL-12/23 inhibitors:Ustekinumab, Gusetuximab; IL-17A inhibitors:Sikuximab, Yiqizhumab	Anti-inflammatory effect, improving skin lesions and enhancing quality of life in patients
Psychotropic drugs therapy	Combination of antidepressants (metoclopramide) and local corticosteroids; Escitalopram (a selective serotonin reuptake inhibitor) combined with psychological intervention treatment	Reduce peripheral cytokine levels, alleviating inflammatory reactions
Psychological intervention therapy	Cognitive Behavioral Therapy (CBT); Meditation Based Mind Body Interventions (MBIs);	CBT: helping patients actively identify and correct past negative beliefs, correct negative thinking and behavior patterns, and reduce psychological burden; MBIs: combines the body and mind to relieve psychological stress, reduce the levels of C-reactive protein and inflammatory cytokines in patients’ bodies
Multidisciplinary diagnosis, treatment, and educational intervention	Diagnosis, treatment and management of patients with psoriasis require the collaborative efforts of multiple disciplines such as dermatology and rheumatology and immunology	Through individual or group intervention, it is possible to effectively improve patients’ treatment compliance, thereby reducing treatment costs and improving disease prognosis.

## 5 Limitations and future investigations

Current research on the comorbidity of psoriasis and depression still has significant limitations across multiple aspects. First, the assessment of comorbid depressive symptoms in patients with psoriasis is notably limited. Specifically, there is high heterogeneity among assessment tools, a lack of standardization, and incomplete assessment domains—most tools primarily focus on the severity of depressive symptoms while omitting critical dimensions such as stigma and cognitive function. Collectively, these issues make it difficult to accurately capture the true nature of this comorbidity. Second, most existing studies suffer from small sample sizes, inadequate population representativeness (e.g., overrepresentation of adult patients and geographic constraints), and a dominance of cross-sectional designs. These factors collectively hinder the drawing of robust conclusions regarding “comorbidity mechanisms” and “causal relationships.” Third, investigations into comorbidity mechanisms remain fragmented: the majority of studies focus on isolated biomarkers (e.g., a single inflammatory cytokine or neurotransmitter) instead of establishing a systematic “multi-pathway integration model.” Additionally, the “peripheral-central” link—connecting core molecular pathways, peripheral inflammation, and central nervous system (CNS) abnormalities—remains poorly defined. Fourth, current intervention studies predominantly target “psoriasis treatment” or “depression treatment” in isolation, with a scarcity of comorbidity-specific intervention strategies. Furthermore, the mechanism underlying how interventions (e.g., biologics) alleviate depression—whether through direct anti-inflammatory effects or indirect alleviation of skin lesion-related psychological distress—remains unclear. To address these limitations, future research should advance in multiple dimensions while enhancing multidisciplinary collaboration and clinical translation. First, researchers should develop a comprehensive assessment framework that integrates “cutaneous pathological features, psychiatric symptoms, and psychosocial factors,” unify assessment instruments, expand assessment domains, and standardize multi-center assessment protocols. This will improve the accuracy and standardization of comorbidity evaluation. Second, researchers should conduct large-scale, diversified, longitudinal cohort studies encompassing individuals of different age groups, ethnicities, and psoriasis subtypes. Long-term follow-up should be implemented to strengthen causal inference and the generalizability of results. Third, core pathways and interaction networks should be identified via multi-omics integrative analysis (genomics, transcriptomics, metabolomics). The “peripheral-central” link should be investigated using functional magnetic resonance imaging (fMRI) and positron emission tomography (PET), while mechanistic validity should be validated through animal models and cellular experiments. These efforts will help construct a systematic comorbidity mechanism model. Fourth, comorbidity-specific precision intervention studies should be designed to compare the efficacy of combination regimens (e.g., “biologics plus antidepressants” and “biologics plus psychological interventions”). At the same time, efforts should be made to explore early prevention strategies for high-risk populations, thereby ensuring the scientific validity and clinical translational value of research findings.

## 6 Conclusion

The relationship between the inflammatory response in psoriasis and depressive states is both intricate and bidirectional. Depressive can activate the HPA axis, stimulating the hypothalamus to secrete CRH. This hormone further regulates immune cells and cutaneous cells, promoting the release of core pro-inflammatory cytokines such as IL-6, IL-17, and TNF-α, ultimately exacerbating the severity of psoriatic lesions and prolonging the chronicity of psoriasis. Conversely, the systemic inflammatory of psoriasis can disrupt the integrity of the blood-brain barrier (BBB), allowing peripheral pro-inflammatory cytokines to infiltrate the central nervous system and induce neuroinflammation. Meanwhile, the persistent itching, pain, and other physical discomforts brought by psoriatic lesions, as well as the social discrimination and social avoidance caused by exposure of the lesions, further accumulate and exacerbate depressive symptoms—forming a mutually reinforcing pathological cycle between the two conditions. Critically, comorbid depression in psoriasis patients is associated with a substantially elevated suicide risk, profound reduction in quality of life, and diminished treatment adherence This underscores the significantly heightened vulnerability of psoriasis patients to developing depression compared to the general population. Consequently, elucidating the shared pathophysiology of psoriasis and depression and developing effective management strategies for this comorbidity are critically urgent research priorities.
